# Rheumatoid arthritis: pathogenesis and therapeutic advances

**DOI:** 10.1002/mco2.509

**Published:** 2024-03-10

**Authors:** Ying Gao, Yunkai Zhang, Xingguang Liu

**Affiliations:** ^1^ Department of Rheumatology Changhai Hospital Naval Medical University Shanghai China; ^2^ Naval Medical Center Naval Medical University Shanghai China; ^3^ National Key Laboratory of Immunity & Inflammation Naval Medical University Shanghai China; ^4^ Department of Pathogen Biology Naval Medical University Shanghai China

**Keywords:** cellular metabolism, epigenetics, pathogenesis, rheumatoid arthritis, therapy

## Abstract

Rheumatoid arthritis (RA) is a chronic autoimmune disease characterized by the unresolved synovial inflammation for tissues‐destructive consequence, which remains one of significant causes of disability and labor loss, affecting about 0.2–1% global population. Although treatments with disease‐modifying antirheumatic drugs (DMARDs) are effective to control inflammation and decrease bone destruction, the overall remission rates of RA still stay at a low level. Therefore, uncovering the pathogenesis of RA and expediting clinical transformation are imminently in need. Here, we summarize the immunological basis, inflammatory pathways, genetic and epigenetic alterations, and metabolic disorders in RA, with highlights on the abnormality of immune cells atlas, epigenetics, and immunometabolism. Besides an overview of first‐line medications including conventional DMARDs, biologics, and small molecule agents, we discuss in depth promising targeted therapies under clinical or preclinical trials, especially epigenetic and metabolic regulators. Additionally, prospects on precision medicine based on synovial biopsy or RNA‐sequencing and cell therapies of mesenchymal stem cells or chimeric antigen receptor T‐cell are also looked forward. The advancements of pathogenesis and innovations of therapies in RA accelerates the progress of RA treatments.

## Graphical abstract

Rheumatoid arthritis (RA) is a chronic inflammatory and autoimmune disease with multisystem involvement, including genetic, epigenetic, metabolic, and immune factors. This review focuses on the importance of recently identified epigenetic and metabolic risk factors for immune dysfunction and pathogenic tissue‐remodeling programs in RA development. Moreover, this review depicts the therapeutic potential of these metabolite and epigenetic targets and their agents for clinical remission and treatment.

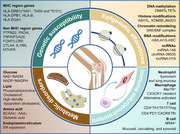

## 1 INTRODUCTION

The descriptions of rheumatoid arthritis (RA) could date back to ancient Greece and Rome, when the term “rheuma” or “rheumatism” were used to describe arthralgia caused by humors imbalance.^1^, ^2^, ^3^ Since 1940, Waaler et al.^4^ discovered that the serum of most RA patients could cause sensitized sheep red blood cells to aggregate and identified rheumatoid factor (RF), the immunological characteristics of RA have been gradually understood. Current recognition is that RA is as an immune system‐mediated chronic inflammatory disease characterized by persistent uncontrollable synovitis, as well as pannus and bone erosion.^5^, ^6^, ^7^, ^8^ Typically, RA manifests as symmetrical polyarthritis, mainly involving small joints of the extremities.^9^, ^10^ Approximately 80% of patients with RA are seropositive, with autoantibodies detectable, such as RF and anticitrullinated protein antibodies (ACPAs).^11^, ^12^ Articular symptoms may be accompanied by systemic complications, including pulmonary interstitial fibrosis, cardiovascular disease, and so on.^13^, ^14^, ^15^ Therefore, when patients accepted substandard or delayed treatments, they might suffer from progressive joints destruction, disability, and even death in the months and years to come.

It's estimated that RA affects 17.6 million people worldwide in 2020, with the age‐adjusted prevalence of 0.21% globally based on Global Burden of Disease (GBD) study, increasing 14.1% than that in 1990.^16^, ^17^ And GBD 2021 RA Collaborators forecasted that there would be 31.7 million RA patients by 2050.^17^ Chinese epidemiological surveys show that there are approximately 5 million RA patients, with a prevalence of 0.42%.^18^ According to cost‐effectiveness analysis, the heavy economic burden brought by RA has long been underestimated.^19^, ^20^

**FIGURE 1:**
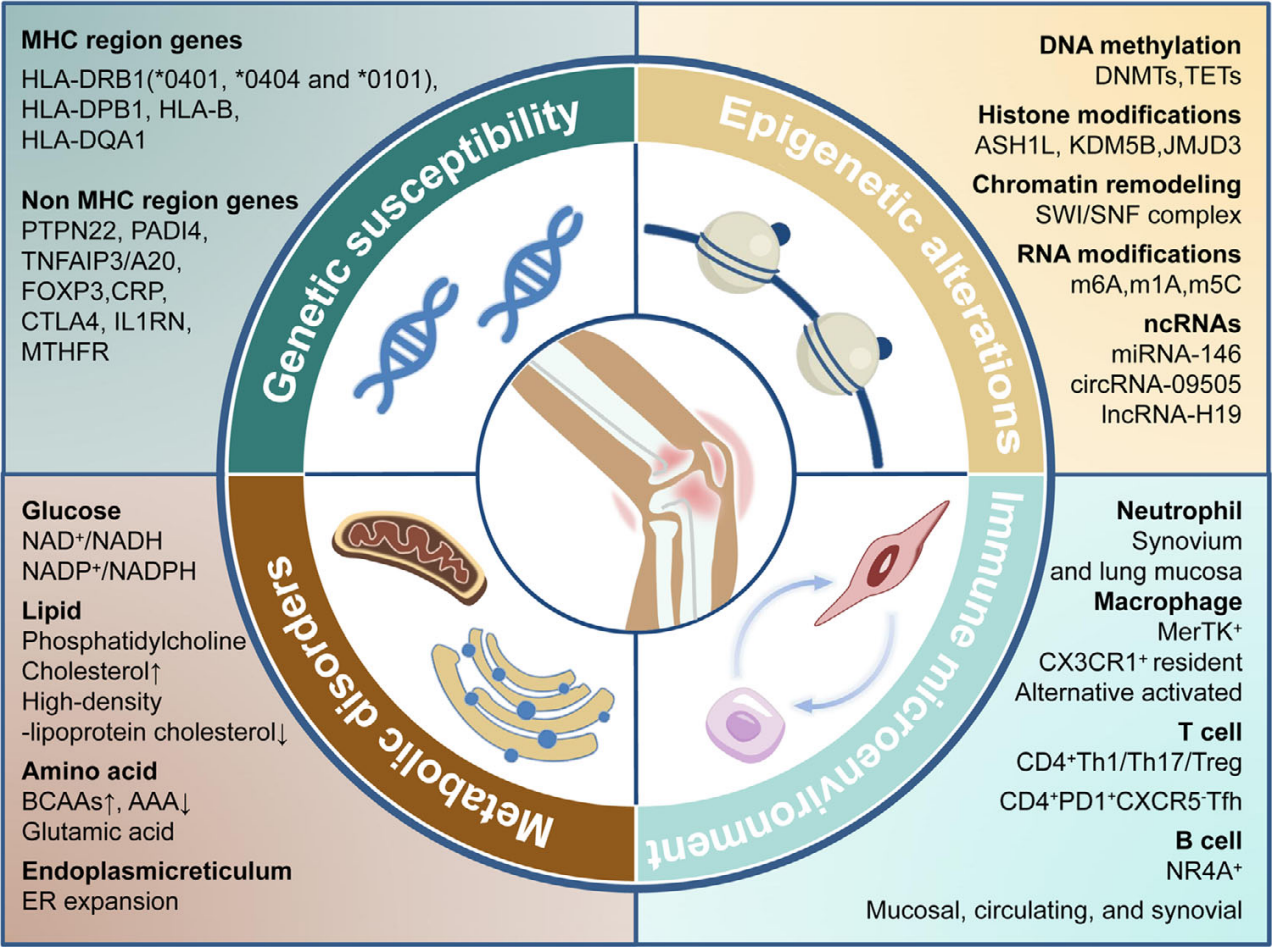
Schematic representation of the pathogenesis of RA. Both MHC region genes and non‐MHC region genes contribute to genetic susceptibility of RA, with HLA‐DRB1 most attributable. Epigenetic alterations participate the development and progress of RA through DNA methylation, histone modification, chromatin remodeling, RNA modification, and ncRNAs. In the immune microenvironment of RA, many pathologic cell subtypes are identified, which might be novel treating targets in the future. Metabolic disorders of glucose, lipid, and amino acid are also found in different cells in rheumatoid joints.

This review is aimed to discuss pathogenesis and therapeutic advances of RA. The well‐known pathogenesis of RA includes production of autoantibodies, mediation of immune cells, activation of inflammatory pathways, and proliferation of synovium. However, these have not offered enough supports for us to seek a cure for RA. Therefore, we will first review recent striking advances in the pathogenesis of RA including immunological, inflammatory, genetic, epigenetic, and metabolic mechanism, with highlights on genetic susceptibility, epigenetic alterations, immune microenvironment, and metabolic disorders, shown in Figure 1. After that, a comprehensive review of available medications for RA is presented. Notably, even though several therapeutic strategies have been proved to be effective for RA, such as biologics and small molecule medications, about 40% patients cannot reach clinical remission. Fortunately, elucidation of uncovered pathogenesis of RA is likely to promote its treatment. Therefore, we also discuss in depth promising targeted therapies under clinical or preclinical trials, especially epigenetic and metabolic regulators. Additionally, prospects on precision medicine and cell therapies are also looked forward.

## 2 PATHOGENESIS OF RA

Even though RA is one of the most common inflammatory arthritis and has been extensively studied as a model for autoimmune diseases for many years, its exact etiology is still unclear. Both genetic and environmental factors promote the susceptibility and onset of RA. Immunological abnormalities, inflammatory pathways, genetic and epigenetic alterations, and metabolic disorders participate in RA pathogenesis, which are mainly summarized here.

### 2.1 Immunological basis of RA

#### 2.1.1 | Role of autoantibodies

Autoantibodies can be detected in RA patient several years before symptoms onset, with ACPAs being the most widely used one in clinical practice. Peptidyl arginine deiminase (PADI) induces the posttranslational modification process of converting arginine or glycine residues of normal proteins into citrulline, which is an important reason for loss of immune tolerance in RA patients.^21^ Citrullinated proteins have a higher affinity for the antigen‐binding groove of human leukocyte antigen (HLA)‐DR and exhibit greater immunogenicity than natural proteins.^22^ Neutrophil extracellular traps (NETs) are an important source of citrullinated proteins. Compared with healthy controls, more NETs were found in the circulation and synovial fluids of RA patients. The formation of NETs leads to the sustained inflammatory microenvironment of the joints.^23^ Research by Carmona‐Rivera et al.^24^ has shown that citrullinated proteins from NETs can be internalized through the receptor for advanced glycation end‐products–Toll‐like receptor 9 (TLR9) pathway, inducing an inflammatory phenotype in fibroblast‐like synoviocytes (FLS) with upregulated membrane major histocompatibility complex II (MHC II) expression. ACPAs not only serve as specific biomarkers reflecting the immune dysfunction of RA, but also play an important role in accelerating the inflammatory response in joints. Furthermore, ACPAs are predictive to bone erosion and cardiovascular diseases as well.

In addition to ACPAs, RFs, anticarbamoyl peptide antibody, autoantibodies against cartilage‐specific antigens such as type II collagen or gp39, and autoantibodies against extracellular antigens of glucose‐6‐phosphate isomerase or heterogeneous nuclear ribonucleoprotein‐A2 are also closely associated with the development and progression of RA.^25^

#### 2.1.2 | Contribution of T cells and B cells

The nonresolving inflammation mediated by aberrantly activated immune cells takes central stage during the pathogenesis of RA. These immune cells diffusely infiltrate synovium, while in others, T cells and B cells cluster in aggregates with or without the support of follicular DCs forming lymphoid aggregates or ectopic germinal centers (GCs),^26^ resulting in the breakdown of self‐tolerance and accelerating RA pathogenesis.

T cells are the dominant lymphocytes infiltrating rheumatoid joints, with a predominance of CD4^+^ T cells over CD8^+^ T cells in most patients.^27^ Synovial T cells have an activated phenotype indicated by its surface markers like CD45, CD44, and MHC II, but their immune responses to stimulation are paradoxically lower than those in control. And interestingly, T cells freshly isolated from rheumatoid synovium behave like cytokine‐stimulated and unlike TCR‐stimulated T cells.^28^ The specific cytokines in inflamed joints induce expression of specific transcription factors, for example, T‐bet and retinoic acid receptor‐related orphan receptor gamma t (RORγT), eventually leading to an imbalance differentiation of T cells, with a preference to helper 1 T cell (Th1) over Th2, and Th17 over regulatory T cell (Treg).^27^

As reported, granzyme K (GZMK)^+^CD8^+^ T cells produce large amounts of interferon (IFN)‐γ in the synovium of RA, bolstering the inflammatory microenvironment.^29^ In addition, recent studies have determined a subset of CD4^+^PD1^+^CXCR5^−^ follicular helper T cells (Tfh) located in proximity to B cells, whose major function is to produce interleukin (IL)‐21 for supporting the proliferation and differentiation of B cells.^30^ Even though B cells only take up a small proportion in the synovium, they are recognized as important participants in the initiation and perpetuation of RA. Activated B cells exert various effector functions, including secretion of inflammatory and regulatory cytokines, formation of ectopic GCs, activation of T cells via antigen presentation as well as costimulatory molecules, and differentiation into antibody‐secreting cells.^31^ B cells and plasmacytes produce RFs and anti‐modified‐protein antibodies (AMPAs), including ACPAs, anticarbamylated protein antibodies and antiacetylated‐protein antibodies. Currently available evidence indicates that most RA patients have at least two kinds of AMPAs, suggesting that a wealth of antigens can potentially activate B cells, both at the time of their initial priming in lymph nodes, and in synovium where they might encounter different modified antigens.^32^

Recently, Meednu et al.^33^ reported that a synovial B cell population characterized by coexpression of nuclear receptor subfamily 4 group A member 1 (NR4A1), NR4A2 and NR4A3, is highly enriched in RA synovial tissue. It supports the formation and functions of ectopic GCs by releasing lymphotoxin α, lymphotoxin β and IL‐6.^33^ Another novel pathogenic B cell subset, called aging‐associated B cells (ABC), has garnered increasing attention. ABCs, initially discovered in systemic lupus erythematosus (SLE), are CD11c^+^ B cells with high expression levels of integrin subunit alpha X (ITGAX), T‐bet, and activation induced cytidine deaminase. Qin et al.^34^ found that these cells were elevated in the synovium and peripheral blood of RA patients and contributed to the pathogenesis of RA by inducing FLS activation.

### 2.2 Inflammatory pathways in RA

#### 2.2.1 | Proinflammatory cytokines

In RA, most proinflammatory cytokines are derived from macrophages and FLS, including tumor necrosis factor (TNF)‐α, IL‐1, IL‐6, and so on. TNF‐α, a major proinflammatory cytokine in RA, is found to amplify inflammation through activating nuclear factor‐kappa B (NF‐κB) pathway, upregulating TNFR II, and inducing receptor activator of NF‐κB ligand (RANKL) secretion in RA‐FLS for osteoclast formation.^35^ Similarly, IL‐1 induces rapid and potent inflammatory responses and is also mainly produced by macrophages in RA. IL‐1 can further stimulate the proliferation of RA‐FLS and the production of IL‐6, IL‐8, GM‐CSF, collagenase, and prostaglandins, and induce the expression of adhesion molecules in RA‐FLS and endothelial cells.^36^ Subsequently, large amounts of IL‐6 in the synovial fluid of RA patients can further transduce pathogenic inflammatory signals in RA‐FLS through the Janus kinase (JAK) pathway, especially the JAK1 and signal transducer and activator of transcription 3 (STAT3) pathway.^37^

However, the levels of Th1 cytokines (IFN‐γ, IL‐2) and Th2 cytokines (IL‐4, IL‐13) are very low and can hardly be detected in the synovium. The content of Th17 cytokine (IL‐17 family) in the synovial fluid is determined to be moderate, but its effects on inflammation and bone destruction are well‐recognized powerful. Especially, the most potent IL‐17A acts directly on FLS, by activating osteoclast activity and promoting bone resorption through upregulating RANKL.^38^

In addition, other inflammatory factors such as IL‐12, IL‐15, IL‐18, IL‐32, GM‐CSF, and chemokines such as IL‐8, monocyte chemoattractant protein‐1 (MCP‐1), and C‐X‐C motif ligand 13 (CXCL13), all contribute partially to RA. Although these cytokines cannot directly lead to RA, they form a complex network with each other, contributing to the pathogenic inflammatory microenvironment within synovium.^39^ The successful clinical application of TNF‐α and IL‐6R antagonists has confirmed the feasibility of cytokine targeted therapy for RA, and exploration of other cytokines targeted medication is ongoing.

#### 2.2.2 Cellular mediators

The hallmark of RA is severe sustained synovitis with marked expansion of synovial lining and sublining layers. There exist two types of synoviocytes in the synovium, namely macrophage‐like synoviocytes and FLS.^40^

Due to the substantial immune functions of macrophage‐like synoviocytes, they are widely accepted as a specific type of immune cell. Circulating monocytes‐derived macrophages, especially those in the lining layer, appear to be a major source of numerous cytokines.^41^ Recently, Kuo et al.^42^ found that heparin‐binding epidermal growth factor‐like growth factor (HBEGF)^+^ macrophages can express NR4A3, urokinase plasminogen activator receptor, and CXCL2, promoting the inflammatory response and invasive behavior of RA‐FLS through epiregulin‐dependent cell‐cell interaction. Hasegawa et al.^43^ performed scRNA‐Seq on C‐X3‐C motif receptor 1 (CX3CR1)^hi^Ly6C^int^ immune cells sorted from the synovium and identified a population of macrophages with osteoclast‐like characteristics. They also found that the differentiation of these specific cells into osteoclasts is mainly regulated by transcription factor forkhead box M1 (Foxm1).^43^ Therefore, macrophages are key regulatory cells in the initiation, expansion, and persistence of RA inflammation. However, not all macrophages are destined to amplify inflammation. Several types of tissue‐resident macrophages have been verified to be protective in RA. For example, MER proto‐oncogene tyrosine kinase (MerTK)^+^ macrophages play an anti‐inflammatory role through the production of lipoxins and resolvins. The expression of MerTK may be induced by growth arrest‐specific protein secreted by CD90^+^ FLS.^44^ Culemann et al.^45^ found that CX3CR1^+^ macrophages, expressing triggering receptor expressed on myeloid cells 2 (Trem2) and V‐set and immunoglobulin domain‐containing 4 (Vsig4), could generate an immune barrier with tight junctions in the sublining layer of the synovium, with strong anti‐inflammatory effects. However, this immune barrier is frequently disrupted in patients with RA.

FLS is derived from the mesenchymal stem cells (MSCs) and exhibits tumor‐like properties, not only proliferating massively but also invading and degrading cartilage.^40^ Other ascribed pathologic functions of FLS include facilitating the differentiation of follicular dendric cells (DCs),^46^ which organize GCs for B cell maturation, and present superantigens to T cells.^47^ With the help of scRNA‐seq technology, several novel types of pathogenic FLS were identified. Wei et al.^48^ recently identified a kind of CD34^−^CD90^+^ FLS emanating from vascular endothelial cells outward by scRNA‐seq of synovial tissue organoids. The differentiation of this subpopulation is indispensable for the development of inflammatory arthritis.^48^ Through longitudinal transcriptomics combined with scRNA‐Seq, Orange et al.^49^ recently identified a kind of CD45^−^CD31^−^PDPN^+^ cell, which exhibits characteristics of FLS, in the peripheral blood of RA patients. This subpopulation was named by PRIME cell. Several weeks before RA relapse, PRIME cells in the peripheral blood are activated by B cells and migrate to the synovial tissue, triggering local inflammation.^49^

**FIGURE 2:**
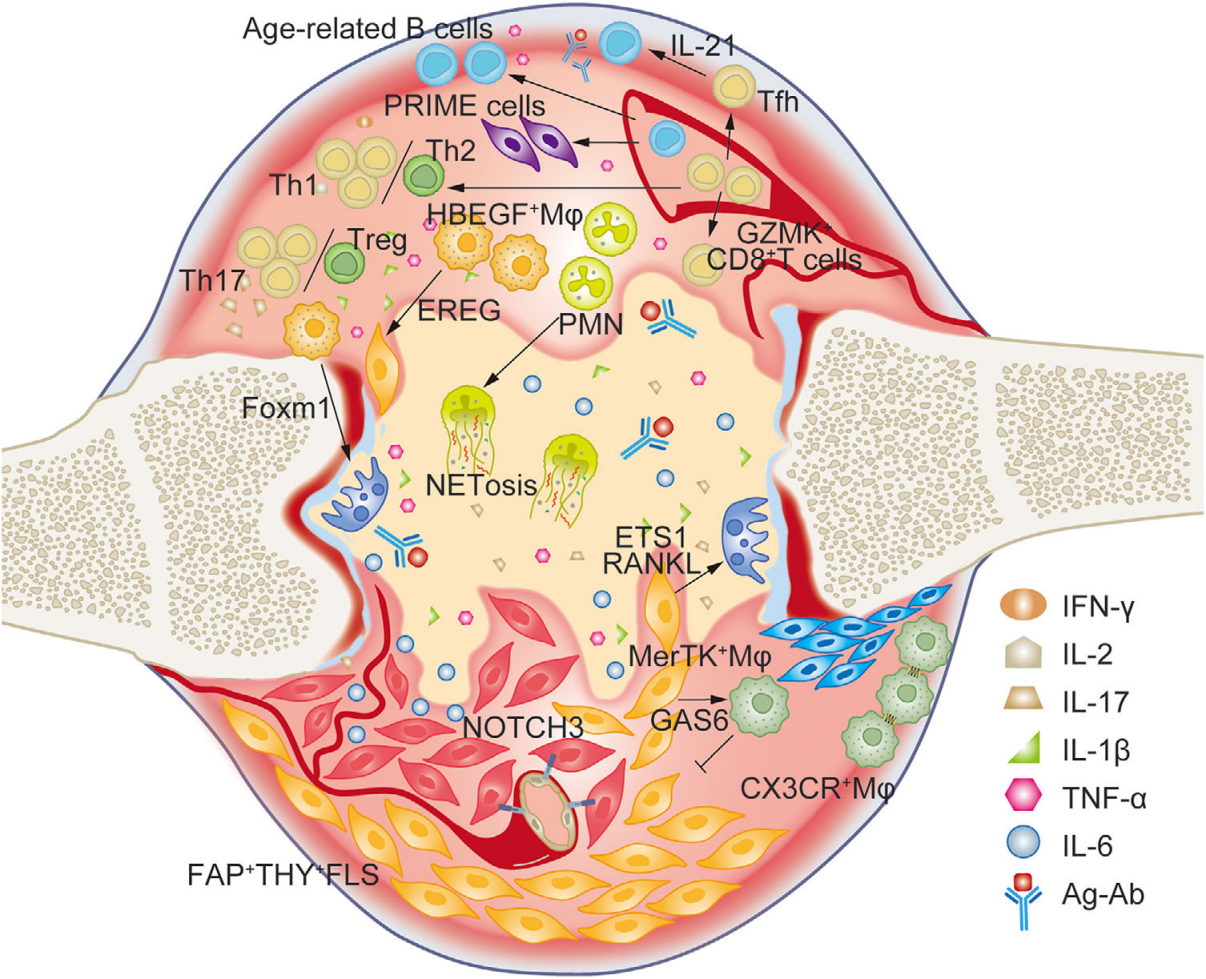
The immune microenvironment composed of different pathogenic or protective cell subsets in RA. The imbalance of Th1/Th2 and Th17/Treg is involved in RA. GZMK^+^CD8^+^ T cells produce large amounts of IFN‐γ; CD4^+^PD1^+^CXCR5^−^ Tfh cells secrete IL‐21 to activated B lymphocytes; PRIME cells derived from B lymphocytes can predict the flare of RA; aging‐associated B cells have been found to be pathogenic in RA; macrophages provide many regulators such as Foxm1 and ETS1 to activate osteoclasts, and EREG to stimulate FLS as well; FAP^+^THY^+^FLS exhibits autoimmune phenotype, and after receiving NOTCH3 signal from endothelial cells, they became invasive. However, some subsets of macrophages are protective, such as MerTK^+^ Mφ and CX3CR^+^ Mφ.

Evaluation of pathogenic cells with scRNA‐seq has provided increasing data on the large array of cell lineages in rheumatoid synovium and peripheral blood mononuclear cell (PBMC),^46^ as shown in Figure 2. These specific cells shed new lights on the pathogenesis of RA, which may serve as potential intervention targets.

#### 2.2.3 Immunomodulatory signaling

In rheumatoid joints, both immune cells and stromal cells recognize various signals and activates corresponding signaling pathways. Abnormal responses in diverse signaling pathways are involved in RA pathogenesis.

##### JAK–STAT pathway

The progression of RA is firstly found to be closely related to highly activated JAK–STAT pathway. Among the four members of the JAK family, JAK2 is the most widely studied protein. Research has shown that JAK2 expression is upregulated in synovial tissues of RA patients.^50^ JAK2 mainly induces the activation of downstream molecules STAT3 and STAT5. Ju et al.^51^ found that activated STAT3 not only promoted rapid proliferation, long‐term survival, tissue invasion, and other tumor‐like characteristics in FLS, but also induced an imbalanced differentiation to Th17 rather than Treg by reducing STAT5. In addition, STAT1 is also upregulated in RA, exerting a relatively stronger stimulative effect on inflammation than on apoptosis of FLS.

##### Mitogen‐activated protein kinase pathway

Almost all mitogen‐activated protein kinase (MAPK) subfamilies participate in RA progression. ERK1/2 regulates the production of IL‐6, IL‐12, IL‐23, and TNF‐α in proinflammatory macrophages,^52^ and regulates the production of COX2‐dependent PGE2 in FLS as well.^53^ IL‐1β and TNF‐α stimulate the activation of JNK, which mainly regulates the expression of matrix metalloproteinase (MMP) in chondrocytes and FLS, promotes the differentiation of monocytes into osteoclasts, and facilitates the degradation of extracellular matrix. Studies have shown that p38 phosphorylation is upregulated by MKK3/6 in synovial tissues of RA, and p38 activation results in the overexpression of chemokines such as IL‐8 and MCP‐1, which facilitate synovial expansion. In addition, p38 induced by integrins leads to abnormal T cell apoptosis, resulting in massive infiltration of synovial tissues and exacerbating the disease process.^54^

##### NF‐κB pathway

In the synovial tissue of RA patients, NF‐κB is highly expressed. The hyperactivation of NF‐κB pathway can induce the expression of inflammatory cytokines such as TNF‐α, IL‐1β, IL‐6, which further amplify NF‐κB signaling to form a vicious cycle.^55^ Excessive activation of NF‐κB can cause not only abnormal apoptosis of FLS for synovial hyperplasia, but also participation in RANKL‐RANK ligation‐mediated osteoclast differentiation, resulting in undesirable bone erosion.^56^

##### Other pathways

The Wnt pathway plays a dual role in RA. Overexpressed Wnt protein, such as Wnt1, Wnt5a, Wnt7b, and Wnt10b, promotes abnormal proliferation and proinflammatory cytokine production in FLS, and is consistent with the degree of inflammatory infiltration and tissue fibrosis. On the other hand, the Wnt pathway also plays a very important role in regulating osteogenic and osteoclastic lineage differentiation.^57^ The Notch signaling is also proved to be involved in the development and progression of RA. Recent studies revealed that Notch‐1 directly bound the promoters of *Il17a* and *Rorc* (encoding RORγT) to enhance Th17 differentiation and function,^58^ and Notch‐3, highly expressed in RA‐FLS, made RA‐FLS invasive to cartilages.^48^

### 2.3 Genetic and epigenetic factors

#### 2.3.1 Genetic susceptibility and risk loci

RA has a strong genetic component, with an estimated a heritability of 53−68%, especially pertaining to patients who are positive for ACPAs.^59^, ^60^ HLA‐DRB1 alleles residing in the MHC region are most attributable, explaining ∼20% of the genetic risk for RA.^60^ The “shared epitope” (SE) hypothesis firstly proposed by Gregersen et al.^61^ in 1987 and the “antigen‐binding groove” hypothesis proposed by Raychaudhuri and coworkers^62^ in 2012 elegantly explained why specific antigen peptides could bind to certain residues of MHC II. A study on the Han Chinese population found that aspartate on position 160 of HLA‐DQA1 significantly increased susceptibility to RA (odd ratio, OR = 2.29).^63^

Many loci from other genes outside the MHC region contribute to RA as well, such as tyrosine‐protein phosphatase nonreceptor type 22 (*PTPN22*)^64^ with an OR of about 1.75^65^ and peptidyl arginine deiminase 4 (*PADI4*)^66^ with an OR of 1.30 per risk allele.^67^ By genome‐wide association studies, over 100 loci across the genome harboring RA susceptibility variants have been uncovered,^68^, ^69^, ^70^, ^71^, ^72^ though most of them are only modestly associated. Here, we displayed in Table 1 several genes and their genetic variants contributing to the susceptibilities of RA, with potential functional relevance.

**TABLE 1:** Single nucleotide polymorphisms (SNPs) in RA and potential functions.

Gene name	SNPs	VDAS	Potential functional relevance	Ancestry	References
HLA‐DRB1	rs660895	0.81	Identification and presentation of modified autoantigens	European or Asian	^73^
PTPN22	rs2476601	1	T cells activation by Csk and Lck	European	^69^
PADI4	rs2240335	0.81	Disturbance of immune tolerance by citrullination of articular peptides	European or Asian	^74^
STAT4	rs7574865	0.9	Abnormal responses to IL‐12 in lymphocytes and regulations of the differentiation of T helper cells	European or Asian	^70^
CTLA4	rs3087243	0.85	Abnormality in inhibitory signal to T cells	European or Asian	^68^
TRAF1‐C5	rs3761847	0.87	Abnormal responses to TNF and downstream signals	European or Asian	^75^
TNFAIP3	rs5029937	0.74	Abnormal responses to TNF and downstream signals	European or Asian	^76^
FOXP3	rs2232365	0.01	Abnormality in the differentiation of Tregs	European or Asian	^77^
CRP	rs756067092	0.01	Disturbance of rapid inflammatory response	European or Asian	^78^
IL1RN	rs2234663	0.01	Abnormality in IL‐1 related immune and inflammatory responses	European or Asian	^79^
MTHFR	rs1217691063	0.1	Interference with one carbon unit metabolism, nucleotide synthesis, lymphocyte proliferation, and MTX‐related toxicity	European or Asian	^80^
IL6R	rs2228145	0.85	Abnormal responses to IL‐6 and downstream signals	European or Asian	^76^
IL1B	rs549858786	0.01	Abnormality in IL‐ 1 related immune and inflammatory responses	European or Asian	^81^
TLR2	rs121917864	0.01	Abnormal responses to PAMPs and downstream signals	European or Asian	^82^
TNF	rs3093662	0.7	Abnormal responses to TNF and downstream signals	European or Asian	^75^
IRF5	rs3807306	0.8	Abnormal responses to IFN and downstream signals	European or Asian	^69^
CDK6	rs4272	0.8	Disturbance of cell cycles	European or Asian	^74^
ARID5B	rs10821944	0.8	Abnormality in the growth and differentiation of B‐lymphocyte progenitors	European or Asian	^83^
TRAF6	rs540386	0.72	Abnormal responses to TNF and downstream signals	European or Asian	^70^
TYK2	rs34536443	0.83	Abnormality in the activation of JAK/STAT signaling pathway	European	^74^
AIRE	rs2075876	0.86	Abnormality in the expression of autoantigens	European or Asian	^84^
CD40	rs4810485	0.84	Abnormal responses to TNF and downstream signals	European or Asian	^74^
CCR6	rs3093024	0.81	Abnormal functions of immature dendritic cells and memory T cells	European or Asian	^74^
CCL21	rs2812378	0.82	Abnormality in homing of lymphocytes to secondary lymphoid organs	European or Asian	^70^
IL2RA	rs2104286	0.83	Abnormal responses to IL‐2 and downstream signals	European or Asian	^69^
IL2RB	rs743777	0.82	Abnormal responses to IL‐2 and downstream signals	European or Asian	^68^
CD244	rs3766379	0.7	Disturbance of NK‐cell cytolytic activity	Asian	^85^
DPP4	rs12617656	0.8	Disturbance of glucose metabolism and immune regulation	Asian	^86^
SFTPD	rs726288	0.8	Disturbance of surfactant metabolism and mucosal immunity	Asian	^87^
ANKRD55	rs6859219	0.81	Unknown	European	^69^

Abbreviations: AIRE, autoimmune regulator; ANKRD55, ankyrin repeat domain 55; ARID5B, AT‐rich interaction domain 5B; C5, Complement Component 5; CCL21, C‐C motif chemokine ligand 21; CCR6, C‐C motif chemokine receptor 6; CDK6, cyclin dependent kinase 6; CRP, C reactive protein; CTLA4, cytotoxic T‐lymphocyte associated protein 4; DPP4, dipeptidyl peptidase 4; FOXP3, forkhead box P3; IL1RN, interleukin 1 receptor antagonist; IL1B, interleukin 1 beta; IL6R, interleukin 6 receptor; IRF5, interferon regulatory factor 5; MTHFR, methylenetetrahydrofolate reductase; MTX, Methotrexate; SFTPD, surfactant protein D; TLR2, Toll‐like receptor 2; TNFAIP3, TNF alpha induced protein 3; TRAF1, TNF receptor associated factor 1; TRAF6, TNF receptor associated factor 6; TYK2, tyrosine kinase 2; VDAS, variants‐disease association scores.

In addition, environmental triggers, such as smoking, exposure to silica dust, low levels of vitamin D, EB virus infection, or periodontitis caused by *P. gingivalis*,^88^ may boost RA risks through gene‐environment interaction. Studies have shown that smokers carrying two RA SEs have a 40‐fold increased risk of RA.^89^ PADI4‐mediated citrullination^90^ and molecular mimicry^91^ partially elucidated this phenomenon.

#### 2.3.2 Epigenetic alterations

Recently, the changes in epigenome provide new insights into RA susceptibility, including DNA methylation, histone modification, chromatin remodeling, and noncoding RNA (ncRNA) (Table 2).

**TABLE 2:** Potential epigenetic targets in RA and biological functions.

Target	Description	Epigenetic factor	Biological functions	References
DNA methylation				
PTEN hypermethylation	Phosphatase and tensin homolog deleted on chromosome ten	DNMT1	FLS activation and proliferation, and release of chemokines and inflammatory cytokines	^92^
TGFBR2 hypermethylation	Transforming growth factor, β receptor II	Unknown	TGF‐β activity and articular cartilage degeneration	^93^, ^94^
STAT3 hypomethylation	Signal transducer and activator of transcription 3	Unknown	IL‐6 pathway in macrophages, T cells and B cells	^94^
FOXP3 hypomethylation	Forkhead box protein P3	Unknown	Regulatory T cell function and the therapeutic effects of MTX	^95^
Histone modifications				
NLRP3	NOD‐like receptor thermal protein domain associated protein 3	KAT2A‐H3K9ac	NLRP3 inflammasome activation, IL‐1β secretion and cell pyroptosis	^96^
PCNA	Proliferating cell nuclear antigen	JMJD3‐H3K27me3	FLS proliferation and migration	^97^
A20/TNFAIP3	Tumor necrosis factor alpha‐induced protein 3	ASH1L/H3K4me3	NF‐κB signal and cytokine production	^98^
IκBα/NFKBIA	Nuclear factor‐kappa‐B inhibitor‐alpha	KDM5B/H3K4me3	Inflammatory macrophage activation	^99^
SFRP1	Secreted frizzled‐related protein 1	EZH2/H3K27me3	Inhibitor of Wnt signaling	^100^
NFATC1	Nuclear factor of activated T‐cells 1	BRD4/Histone acetylation	Osteoclastogenesis and TNF‐induced bone resorption	^101^
CSF3	Colony stimulating factor 3 (granulocyte)	UHRF1	FLS apoptosis resistance and upregulated expression of cytokines	^102^
Transcription factors				
STAT3	Signal transducer and activator of transcription 3	JMJD1C	B cell differentiation and antibody‐mediated autoimmunity	^103^
ETS1	ETS Proto‐Oncogene 1	H3K27ac	RANKL and matrix metalloproteinases production in FLS	^104^
NF‐κB	Nuclear factor‐kappaB	USP7	Toll‐like receptor‐induced proinflammatory cytokine expression	^105^
RNA modifications				
PGC‐1α	Peroxisome proliferator‐activated receptor gamma coactivator 1α	METTL3	Mitochondrial dysfunction and oxLDL‐induced inflammation	^106^
A20/TNFAIP3	Tumor necrosis factor alpha‐induced protein 3	METTL14	NF‐κB signal and cytokine production	^107^
JARID2	Jumonji AT rich interacting domain 2	ALKBH5/IGF2BP3	Proliferation, migration, and invasion of RA FLS	^108^
Chromatin remodeling and architecture				
ARID5B	AT‐rich interactive domain‐containing protein 5B	CTCF	Development and function of T cells and B cells	^109^
ST6GAL1	β‐Galactoside α−2,6‐sialyltransferase 1	CTCF	Sialylation of anticitrullinated protein antibodies	^110^
IL‐6	Interleukin‐6	BRG1‐KDM2B	IL‐6 production and effect	^111^

Abbreviations: ALKBH5, AlkB Homolog 5; ASH1L, ASH1 like histone lysine methyltransferase; BRD4, bromodomain containing 4; BRG1, Brahma‐related gene‐1; CTCF, CCCTC‐binding factor; DNMT1, DNA methyltransferase 1; EZH2, Enhancer of zeste homolog 2; HDAC6, histone deacetylase 6; IGF2BP3, insulin like growth factor 2 mRNA binding protein 3; JMJD1C, Jumonji domain containing 1C; JMJD3, Jumonji domain containing‐3; KAT2A, lysine acetyltransferase 2A; KDM2B, lysine demethylase 2B; KDM5B, lysine demethylase 5B; METTL3, methyltransferase‐like 3; METTL14, methyltransferase‐like 14; UHRF1, ubiquitin like with PHD and ring finger domains 1; USP7, deubiquitinase ubiquitin‐specific peptidase 7.

##### DNA methylation

DNA methylation is the most widely studied epigenetic modifications in diverse autoimmune diseases. DNA methyltransferase (DNMT) and demethylase ten‐eleven translocation protein (TET) regulate the methylation degree of cytosine‐guanine dinucleotides (CpG) islands, most of which are located in the promoter regions.^112^ Abnormal hypermethylation of the CpG islands prevents the binding of transcription factors to the promoter regions and leads to gene transcriptional expression.^113^ Previous studies have demonstrated the widespread DNA hypomethylation in PBMC of RA patients,^114^ which may lead to the increased expression of proinflammatory cytokines, such as IL‐6.^115^ Hypermethylation of a specific region in the promoter of CTLA‐4 limits the activation of immunomodulatory pathway in Treg.^116^ The methylation pattern of PBMC can be used to anticipate the evolution of RA.^117^

**FIGURE 3:**
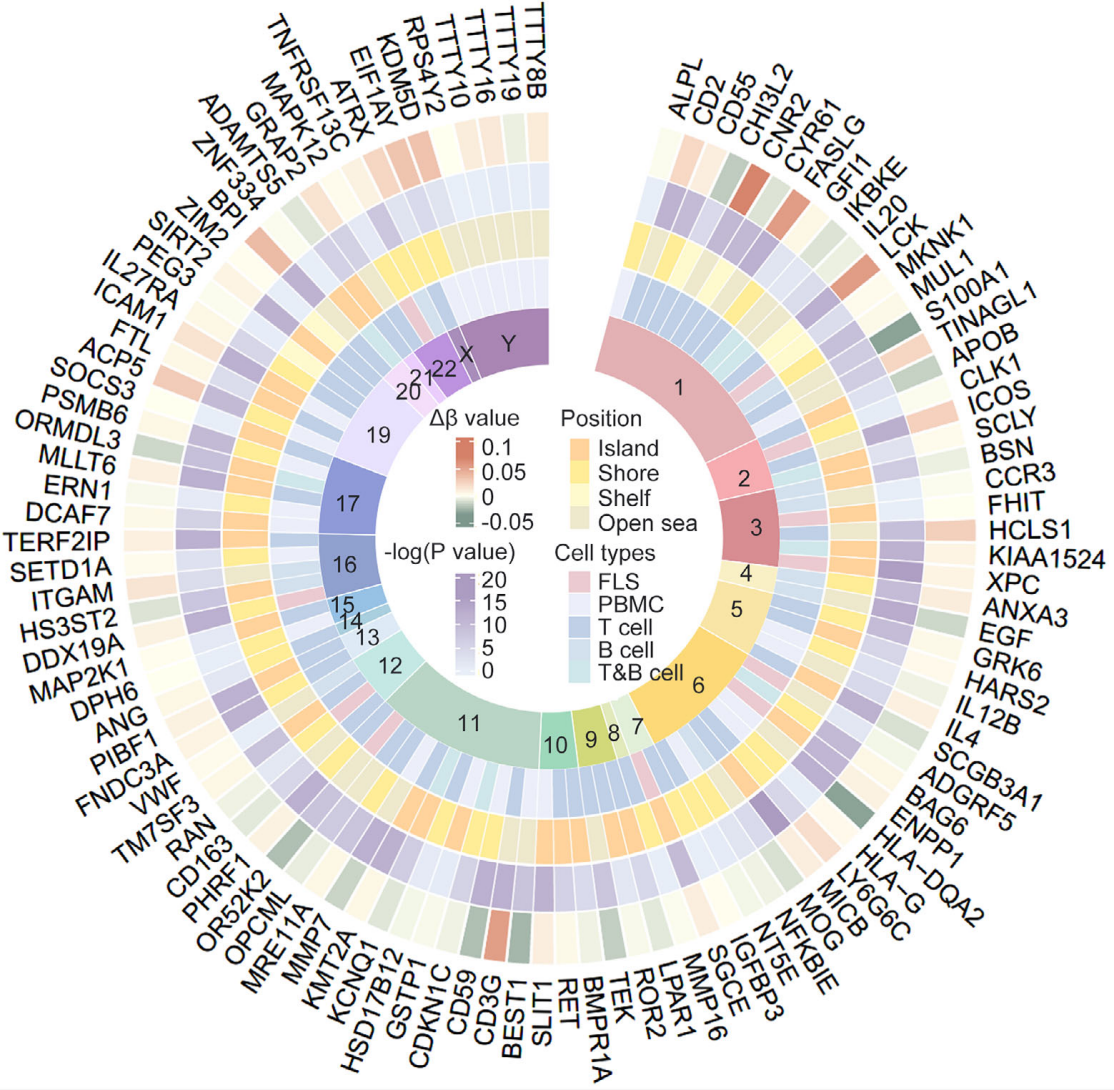
Genes with differential DNA methylation in the promoter regions from DiseaseMeth (V2.0) and EWAS Atlas databases. Δ*β* value represents the degree of DNA methylation. −log(*p* value) stands for a statistical significance. Positions offers detailed information about how far the methylated sites are away from CpG islands. And the specific cell types where DNA methylation happens are presented as well.

Surprisingly, there is no difference in the overall DNA methylation levels of FLS compared to healthy controls.^94^ However, when focused on the promoter regions, it is uncovered that, the percent of hypermethylated CpG sites in gene promoters of FLS increased from 9% in normal controls, to 84% in very early RA and 96% in established RA.^100^ The promoter hypermethylation of specific genes might cause FLS expansion and chronicity of RA. For example, phosphatase and tensin homolog deleted on chromosome ten (PTEN), which inhibits abnormal cell proliferation, is downregulated in RA‐FLS due to DNA hypermethylation in the upstream region of the first exon mediated by DNMT1.^92^ 5‐azadC, a DNA methylation inhibitor, is proven to reduce the release of chemokines and inflammatory cytokines, inhibit FLS activation and proliferation, and alleviate inflammation and damage of joints in animal model of adjuvant‐induced arthritis.^92^ Several studies utilizing whole genome bisulfite sequencing or other DNA methylation chips have identified many differential DNA methylation sites in RA.^117^, ^118^, ^119^, ^120^, ^121^ We searched the DiseaseMeth (v2.0) and EWAS Atlas databases and conducted a combined analysis of genes with significant differential methylation in the promoter regions of FLS, PBMC, T, and B lymphocytes, as shown in Figure 3.

##### Histone modification

Histone undergoes a variety of posttranslational modifications that bring about proinflammatory gene regulation. Almost all types of modifications, including acetylation, methylation, and ubiquitination, can occur at specific lysine residues on histone tails, among which acetylation is the most common one.^122^ The acetylation of histone 3 lysine 9 (H3K9), H3K14, H3K27, H4K5, H4K16 enhances DNA accessibility and promotes gene transcription under the reversible catalysis by histone deacetylases (HDACs) and histone acetyltransferases (HATs).^122^ Studies on the role of HDACs in RA‐FLS has yielded contradictory results. Huber et al. reported that RA‐FLS exhibits a shift towards high acetylation of histones exactly as RA‐PBMC does,^123^ owing to the decreased activity and expression of HDACs,^124^ while Kawabata et al.^125^ reported the opposite. The confusing phenomenon probably resulted from the different levels of TNF‐α of the enrolled patients.^126^ Anyway, it is unequivocal that TNF promotes the activity of HDACs in RA,^125^ and both selective and nonspecific HDAC inhibitors can alleviate inflammation in arthritis animal models.^126^ Expression and activity of HATs in synovial tissue from RA also remains unclear. Our previous research elucidated that KAT2A was overexpressed in RA synovium, and pharmacological inhibition of KAT2A significantly alleviated inflammation in collagen‐induced arthritis (CIA) mice.^96^

What is more, the dynamic balance of histone lysine methyltransferases (KMT)/histone lysine demethylase (KDM) regulates the methylation of H3K4, H3K36, and H3K79 to promote gene transcription and the methylation of H3K9, H3K27 and H4K20 to suppress gene transcription.^122^ Studies identified an upregulation of twelve KMTs and four KDMs in RA‐FLS, leading to significant changes in histone methylation patterns.^127^ JMJD3, also named as KDM6B, is upregulated in platelet‐derived growth factor‐stimulated FLS, leading to demethylation of H3K27me3 in the promoter regions of TLR2 and proliferating cell nuclear antigen (PCNA). Subsequently actively transcribed TLR2 and PCNA promote FLS proliferation and migration.^97^ JMJD3‐specific inhibitors could significantly ameliorate autoimmune responses in CIA mice.^128^ EZH2 is an important methyltransferase that catalyzes H3K27me3 modification. EZH2 is proved highly expressed in TNF‐α‐stimulated FLS. EZH2 could downregulate the expression of secreted frizzled‐related protein 1 (sFRP1), a natural Wnt pathway inhibitor, resulting in excessive proliferation of FLS.^100^ In addition, EZH2 is also highly expressed in CD4^+^ naive T cells of RA patients. EZH2 could interfere with Treg differentiation by downregulating mothers against decapentaplegic homolog 7 (SMAD7), thus leading to uncontrollable inflammation in RA.^129^ Our previous research identified the participation of two epigenetic factors in RA pathogenesis. Ash1l enhanced A20 expression through induction of H3K4 modification at the *Tnfaip3* (encoding A20) promoter.^98^ KDM5B selectively bound and mediated the H3K4me3 modification erasing of the promoter of *Nfkbia*, the gene encoding IκBα.^99^ They both regulate NF‐κB‐dependent cytokine production and immune dysregulation in proarthritis macrophages and dendritic cells.

**FIGURE 4:**
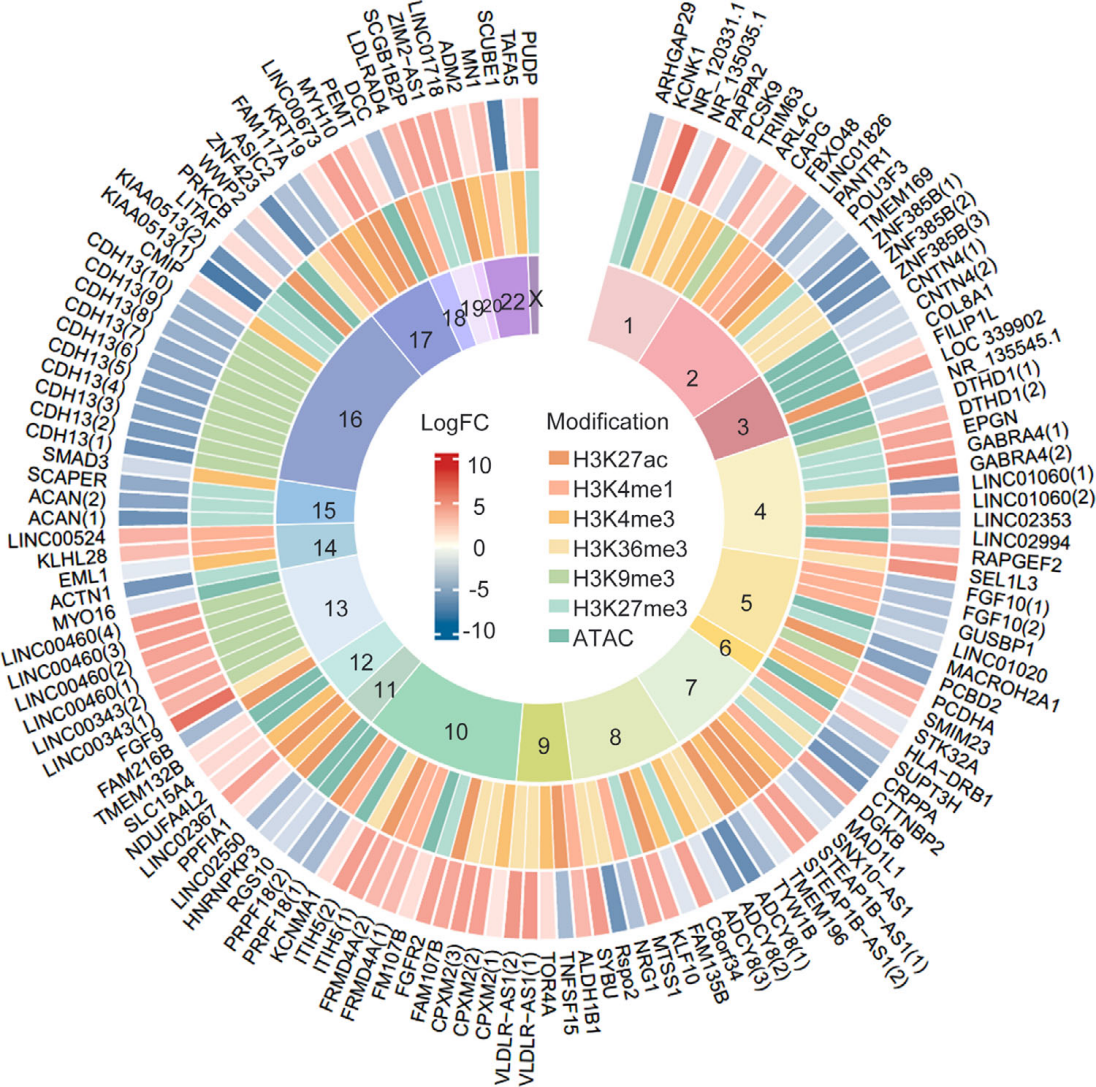
Differentially modified epigenetic regions determined by ChIP‐seq of H3K27ac, H3K4me1, H3K4me3, H3K36me3, H3K9me3, H3K27me3, and ATAC‐seq. Displayed are top 10 genes with differentially modified histones including H3K27ac, H3K4me1, H3K4me3, H3K36me3, H3K9me3, H3K27me3, and chromatin accessibility. LogFC represents the degree of a specific modification.

In 2018, Ai et al.^130^ studied the comprehensive epigenomic characteristics of FLS, identifying nearly one million differentially modified epigenetic regions (DMERs) between RA‐FLS and osteoarthritis‐FLS. However, further research is still needed to understand the function of each DMER. Figure 4 displays the top 10 upregulated DMERs and top 10 downregulated DMERs of six kinds of histone modifications and chromatin accessibility determined by chromatin immunoprecipitation (ChIP) and assay for transposase‐accessible chromatin (ATAC) with high‐throughput sequencing.^130^

##### Transcription factor

A variety of important transcription factors participate in the pathogenesis of RA. Yin et al.^103^ reported that Jumonji domain containing 1C (JMJD1C), which were negatively associated with plasma cell frequency and disease severity in RA, demethylated STAT3 to restrain plasma cell differentiation and pathogenic immunoglobulin production. Yan et al.^104^ identified E26 transformation specific‐1 (ETS1) drove the pathological tissue‐remodeling programs in disease‐associated FLS by orchestrating a previously undescribed regulatory elements (168 kb upstream of the *TNFSF11* transcription start site) of the osteoclast differentiation factor RANKL. Fibroblast‐specific ETS1 deletion resulted in ameliorated bone and cartilage damage under arthritic conditions. In addition, Mitxitorena et al.^105^ uncovered that USP7 stabilized DNA‐bound NF‐κB by opposing the activities of E3 ligases, and thereby promoted NF‐κB‐mediated transcription of proinflammatory cytokines.

##### RNA modifications

RNA modification is one of the new hotspots in the field of epigenetic research. And various RNA modifications have been discovered in recent years, among which RNA 6‐methyladenine (m^6^A) is most widely investigated. Zhang et al. identified that during oxidized low‐density lipoprotein (oxLDL)‐induced monocyte inflammation, METTL3 modified peroxisome proliferator‐activated receptor gamma coactivator 1‐alpha (*PGC‐1α*) mRNA, mediating its RNA degradation, and thereby enhancing the inflammatory response.^106^ Tang et al.^107^ reported a novel mechanism by METTL14‐mediated inhibition of *TNFAIP3* expression via regulation of mRNA stability and translocation in TNFAIP3 protein‐coding regions through m6A modification. They also reported that METTL14 and m^6^A levels were decreased in PBMCs of active RA patients. Conversely, an eraser of m^6^A, ALKBH5 was upregulated in FLS and synovium from RA as reported. ALKBH5 mediated m6A modification in the Jumonji/ARID domain‐containing protein 2 (*JARID2*) mRNA and enhanced its mRNA stability, promoting the proliferation, migration, and invasion of RA‐FLS.^108^

##### Chromatin remodeling and architecture

The degree of chromatin compression, also known as chromatin accessibility, is one of the key factors allowing a physical contact between transcription factors or RNA polymerase II with regulatory elements such as promoters and enhancers. Jadhav et al. reported that gene regulatory sites with more chromatin accessibility in peripheral CD4^+^ T cell from RA patients were highly enriched for the motif of the CTCF, whereas other sites with reduced chromatin accessibility were enriched for motifs of TFs pertinent for T cell function.^109^ In another research, CTCF was also the specific transcription factor of β‐galactoside α−2,6‐sialyltransferase 1 (ST6GAL1) in B cells, which upregulated the sialylation of ACPAs in RA and attenuated the disease progression.^110^

##### noncoding RNA

Large numbers of studies demonstrate that ncRNAs contribute to the pathogenesis of RA by regulating the expression of specific target genes.^112^ For example, miR‐146 is upregulated in FLS of RA patients and is closely related to the disease activity of RA.^131^ Overexpression of miR‐203 leads to hypomethylation of the *Il6* gene promoter regions,^132^ causing IL‐6‐dependent inflammation and tissue damage. Long ncRNAs (lncRNA) and circular RNAs (circRNA) usually exert their functions through miRNAs. For instance, LncRNA H19 sponges miR‐103a, which negatively regulates IL‐15 and Dickkopf‐related protein 1 (DKK1) in RA‐FLSs.^133^ LncRNA Nuclear Enriched Abundant Transcript 1 (NEAT1) sponges miR‐410‐3p, which negatively regulates YY1 in RA‐FLSs,^134^ and sponges miR‐23a, which negatively regulates the murine double minute‐2 (MDM2)–sirtuin 6 (SIRT6) axis in RA‐PBMCs.^135^ Circ_0088036 sponges miR‐140‐3p, which negatively regulates sirtuin 1 (SIRT1) in RA‐FLSs.^136^ Circ_09505 sponges miR‐6089, which negatively regulates AKT1 in RA‐PBMCs.^137^ ncRNAs are biologically important, owing to their cell‐ and tissue‐type specificity, especially in pathogenic cells, joint tissues and biofluids, thus having been explored as potential biomarkers, mediators of pathogenesis, and therapeutic targets.

### 2.4 Metabolic disorders of RA

**FIGURE 5:**
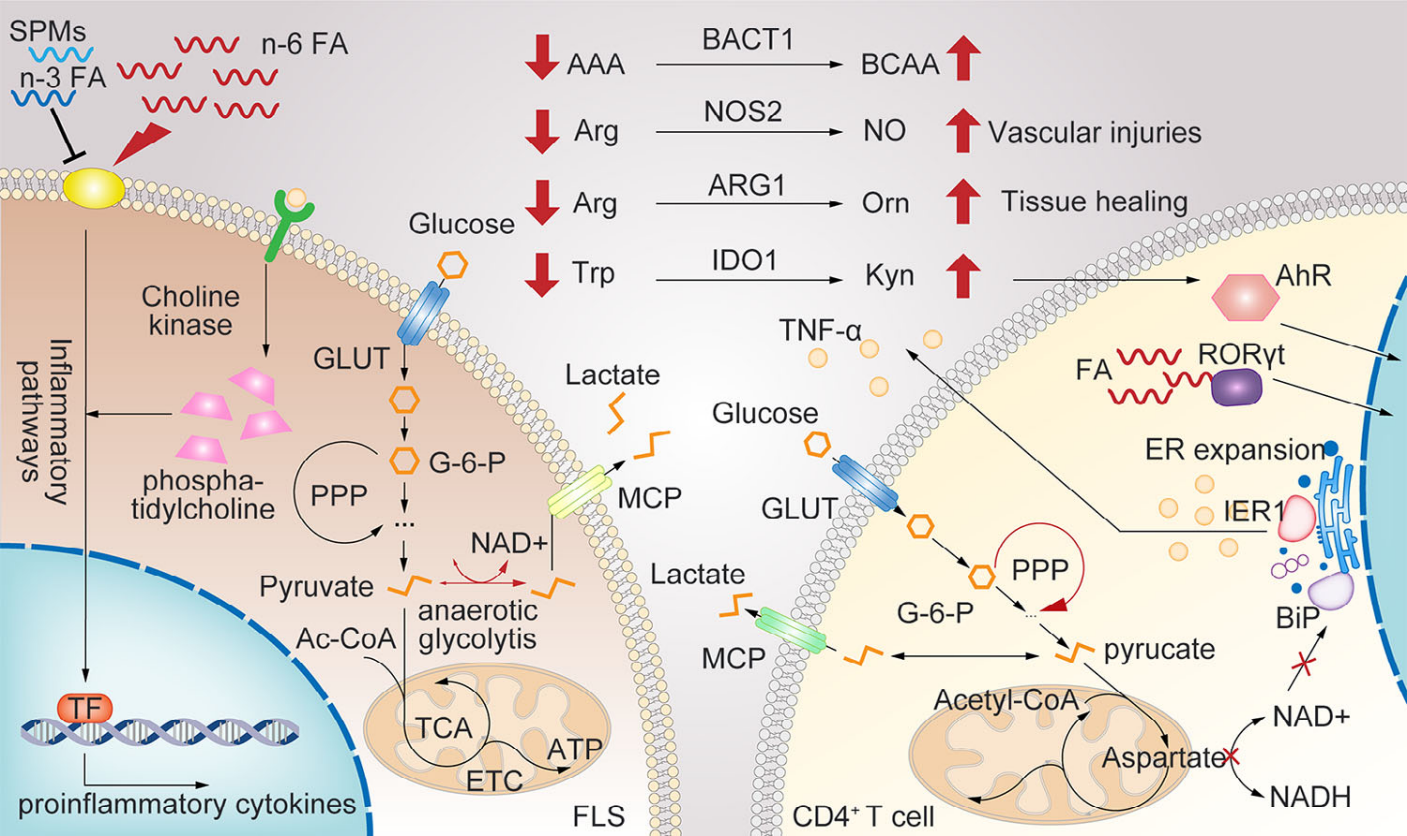
Immunometabolism alterations in RA. Red lines and arrows represent abnormal metabolic changes. CD4^+^ T cells in RA shift from glycolysis to PPP, while anaerobic glycolysis in RA‐FLS increases, resulting in excessive lactate production. Depolarization and dysfunction of mitochondrial in T cells in RA are related to ER expansion and TNF‐α synthesis. n‐6 Polyunsaturated fatty acids (PUFA) and its derivatives are mainly proinflammatory, while n‐3 PUFA and specialized proresolving mediators (SPMs) play important roles in the regression of inflammation. The derivatives of cholesterol and FAs are also the natural ligand of RORγt. In addition, TNF‐α can upregulate choline kinase and phosphatidylcholine levels in RA‐FLS. The levels of BCAAs increase while AAAs decrease in RA patients. Blocking BCAT1 reduces the severity of RA. Arginine (Arg), tryptophan (Trp), and their key enzyme in metabolic pathways participate in AhR activation, vascular injuries, and tissue healing.

In recent years, the mechanisms of energy metabolism in rheumatic diseases have received high attention from researchers. The six major metabolic pathways, namely glycolysis, tricarboxylic acid cycle, pentose phosphate pathway (PPP), fatty acid oxidation, fatty acid synthesis, and amino acid metabolism, respectively, play important roles in several parts of RA progression, including synovial cell activation, proliferation, and differentiation. Different types of cells take advantage of different metabolic pathways for their diverse functions, and abnormal cellular metabolic changes promote autoimmune diseases including RA (Figure 5).^138^ Intervening the metabolic state of cells to regulate their function may provide new perspectives for disease management.

#### 2.4.1 Glycometabolism

In the early stage of RA, glucose metabolism in CD4^+^ T cells shifts from classical glycolysis to the PPP. The activity of 6‐phosphofructo‐2‐kinase/fructose‐2,6‐biphosphatase 3 (PFKFB3) in peripheral blood CD4^+^ T cells decreases, leading to reduced ATP and lactate production, thus enhancing an influx to PPP and production of a large amount of NADPH and 5‐phosphoribose. Increased NADPH can neutralize reactive oxygen species (ROS), which is necessary for T cell activation. ROS acts as a second messenger to alter multiple signaling pathways, resulting in activation and cut‐down of G2‐M phase by inhibiting the activity of cyclin‐dependent kinases. This leads to excessive proliferation of T cells and differentiation into pathogenic Th1 and Th17 cells other than Tregs.^139^

In the late stage of RA, due to tissue hypoxia and mitochondrial dysfunction in synovial cells, anaerobic glycolysis increases, resulting in excessive lactate production. The acidic environment affects immune cell function, such as inhibiting IL‐17A production by CD4^+^ T cells and affecting the cytolytic function of CD8^+^ T cells. In addition, metabolic changes in synovial cells are directly related to cell damage and inflammatory cascades. Studies have shown that hypoxia in synovial tissues of RA patients is accompanied by increased expression of glycolytic enzymes. Overexpression of hexokinase 2 in RA‐FLS enhances cell migration and invasion ability. Increased lactate participates in maintaining the angiogenic capacity for a guarantee of tissue oxygen uptake, mitochondrial integrity and ATP production.^140^

In addition, the study by Wu et al.^141^ showed that the depolarization and dysfunction of mitochondrial in T cells from RA patients were related to endoplasmic reticulum (ER) expansion. The size of ER in T cells is directly related to TNF‐α synthesis and secretion, with significantly higher ER content in T cells from RA patients compared with normal controls. The altered oxidative metabolism in T cells derived from RA patients leads to decreased abundance of aspartate. Aspartate usually shuttles from mitochondria to cytoplasm, where it is converted back to oxaloacetate and participates in cytoplasmic regeneration of nicotinamide adenine dinucleotide (NAD^+^). Defective aspartate shuttle leads to reduced levels of NAD^+^, reducing ADP‐ribosylation of NAD^+^‐dependent proteins. ER molecular chaperone binding immunoglobulin protein (BiP), without ADP‐ribosylation, can release ER stress proteins (e.g., Inositol‐requiring enzyme‐1α), thereby driving ER expansion and transforming T cells into TNF‐α super‐producers.^141^

#### 2.4.2 Lipid metabolism

Metabolomics studies have revealed increased levels of cholesterol in the sera of RA patients, and decreased levels of high‐density lipoprotein cholesterol.^142^ Therefore, researchers have considered lipid metabolism as a checkpoint for cardiovascular complication in RA. Fatty acid (FA) changes in RA patients are also often detected in clinical tests. Lipid reprogramming affects proinflammatory signaling cascades by affecting membrane fluidity and lipid raft formation. Arachidonic acid in n‐6 polyunsaturated fatty acids (PUFA) and its derivatives are mainly considered as proinflammatory mediators, while eicosapentaenoic acid (EPA) and docosahexaenoic acid in n‐3 PUFA are considered as mediators inducing inflammation resolution. In addition, specialized proresolving mediators (SPMs), including lipoxins, E‐series resolvins, D‐series resolvins, protectins, and maresins, also play important roles in the regression of inflammation. In sera and synovial fluid of RA patients, both higher level of leukotriene B4 (LTB4) derived from arachidonic acid and lower level of SPMs are observed.^143^

Mitochondrial dysfunction caused by inactivation of AMP‐activated protein kinase redirects T cell metabolism from FA oxidation to FA synthesis, promoting cytokine production and T cell infiltration into joints.^144^ In addition, the derivatives of cholesterol and FAs are also the natural ligand of RORγt, the master transcription factor for IL‐17 production.^145^ In FLS, free FA dose‐dependently increases the secretion of proinflammatory cytokines, chemokines, and matrix degrading enzymes. For example, palmitic acid (PA) can induce the secretion of IL‐6 in FLS.^146^ RA‐FLS also exhibits active choline metabolism for the expression of phosphatidylcholine, with choline kinase being a crucial enzyme in the cytidine diphosphate–choline pathway. TNF‐α can upregulate choline kinase and phosphatidylcholine levels in RA‐FLS, suggesting abnormal activation of this metabolic pathway by the inflammatory synovial microenvironment during RA. Therefore, blocking choline kinase can inhibit the invasive phenotype of RA‐FLS.^144^ Furthermore, increasing FA oxidation can induce osteoclast precursors fusion and promote RA joint destruction, including PA, LTB4 and lysophosphatidic acid.^147^ On the contrary, docose hexaenoie acid inhibits MAPK and NF‐κB pathway of T cells and accelerates the apoptosis of mature osteoclasts by inducing the expression of Bim.^148^

#### 2.4.3 Amino acid metabolism

It has been suggested that the levels of branched‐chain amino acids (BCAAs) increase, while aromatic amino acids (AAAs) decrease in RA patients, which may be related to changes in immune cell activity and inflammatory mediators.^149^ Papathanassiu et al.^150^ reported that blocking branched‐chain aminotransferases (BCAT1) reduced the severity of CIA model by controlling metabolic reprogramming of macrophages.

Overall amino acid levels also alter in the plasma of RA patients, with alanine, histidine, arginine (Arg), valine, serine, tryptophan (Trp), lysine, glycine, arginine and creatinine decreased significantly, and with glutamic acid, kynurenine (Kyn) and homoserine increased markedly.^149^ Indoleamine 2,3‐dioxygenase 1 (IDO1), a key rate‐limiting enzyme of the kynurenine pathway, is associated with RA. Transforming Trp into Kyn, IDO1 suppresses T cell responses by Trp depletion, and dampens immune responses by activating aryl hydrocarbon receptor (AhR) through Kyn.^151^, ^152^ Arg is the substrate of nitric oxide synthases (NOS) and arginases. NOS2 in M1 macrophages transforms Arg into nitric oxide (NO) and l‐citrulline for inflammatory responses, whereas ARG1 activity in M2 macrophages mainly leads to Arg starvation and thus immunoregulatory effects for tissue healing.^153^ Available data indicate that arginases are overexpressed in immune cells of RA patients attempting to refrain inflammation, while the increased consumption of Arg by arginases may significantly reduce the level of NO and increase the risk of cardiovascular symptoms associated with RA.^149^

## 3 ADVANCES IN RA THERAPY

With a deeper understanding of RA pathogenesis, large number of innovative and effective therapeutic drugs have emerged in recent years, leading to significant relief of joint inflammation and bone destruction for RA patients, although RA cannot be cured. Despite that nonsteroidal anti‐inflammatory drugs and steroids have good efficacy in relieving pain and inflammation, they perform poorly in slowing down radiographic progression and may cause a series of adverse events during long‐term use. On the other hand, disease‐modifying antirheumatic drugs (DMARDs), which can effectively prevent joint destruction and disability, remains to be the cornerstone of RA treatment.^154^ The existing DMARDs drugs mainly include the following three categories: conventional synthetic DMARDs (csDMARDs), biological DMARDs (bDMARDs), and targeted synthetic DMARDs (tsDMARDs).

### 3.1 | Traditional DMARDs

Conventional synthetic DMARDs work slowly but can continuously relieve patient disease activity, fundamentally inhibit progressive damage to tissues and joints, and slow down or prevent disease progression. So far, csDMARDs are still the core and mainstay of RA treatment.

#### 3.1.1 Methotrexate and sulfasalazine

Methotrexate (MTX) plays a crucial role in RA treatment and is the first‐line initial treatment for RA. The ACR/EULAR guidelines recommend that all RA patients should use MTX in sufficient doses as early as possible unless MTX intolerance exists. Mechanically, MTX mediates the resolution of inflammation by upregulating adenosine and inhibiting methylation reactions.^155^ Sulfasalazine (SSZ) is a conjugate compound with both anti‐inflammatory effects of 5‐aminosalicylic acid and antibacterial effects of sulfapyrimidine, exerting multiple anti‐inflammatory effects in vitro. SSZ is often used as one of the combination therapies for RA.^156^

#### 3.1.2 Hydroxychloroquine and leflunomide

Hydroxychloroquine (HCQ), a well‐tolerated antimalarial drug, is also commonly used in combination therapy for RA. HCQ has widely accepted immunomodulatory and anti‐inflammatory effects. Previous studies have indicated that HCQ, which is weakly alkaline, can change the pH of lysosomes and ER, leading to decreased protein digestion and antigen presentation ability of monocytes/macrophages.^157^ Generally, HCQ is quite safe, despite of potential eye toxicity for the elderly and long‐term users. Leflunomide (LEF) might be the most widely used substitute of MTX in China. It inhibits the activation and proliferation of T lymphocytes by targeting dihydroorotate dehydrogenase (DHODH). In addition, LEF can inhibit tyrosine kinase phosphorylation, block NF‐κB activation, and enhance the expression of TGF‐β under high concentration.^158^

### 3.2 Biologic DMARDs

Several large‐molecule drugs synthesized by cells have been designed, produced, and been applied to target the overexpression of proinflammatory cytokines and aberrant activation of immune cells in RA. Clinical trials have demonstrated that biologics targeting TNF‐α, IL‐6, CD20, and CTLA‐4 can rapidly improve RA symptoms and clinical signs, and effectively prevent disease progression.

#### 3.2.1 TNF‐α inhibitors

TNF‐α inhibitors are the most widely utilized medications for RA around the world. Clinical trials have shown that TNF‐α inhibitors are more effective in treating RA than MTX monotherapy.^159^ The pharmacological mechanism of TNF‐α inhibitors is to decrease inflammatory mediators such as IL‐1, IL‐6, IL‐8, MMPs, to downregulate VEGF for endothelial cell‐specific angiogenesis, and to reduce the migration of lymphocytes and macrophages into the joints.^160^, ^161^ Therefore, short‐term use of TNF‐α inhibitors can relieve inflammation, and more importantly long‐term use delay bone erosion. The five approved TNF‐α inhibitors for clinical use, including etanercept, infliximab, adalimumab, golimumab, and certolizumab pegol exhibit different characteristics in clinical use.^162^

#### 3.2.2 IL‐6 inhibitors

IL‐6 plays a crucial role in inflammation and immune responses. Tocilizumab is a humanized monoclonal antibody against IL‐6R, which can effectively inhibit a series of reactions induced by IL‐6.^163^ Multiple clinical studies have shown significant efficacy of tocilizumab in patients with refractory RA, and it is also beneficial for the progression of radiographic joint damage assessed by the modified Total Sharp Score (mTSS).^164^ In addition, another IL‐6R human monoclonal antibody, Sarilumab, and an IL‐6 monoclonal antibody, Sirukumab, also shown therapeutic effects in RA, as demonstrated in the MOBILITY study.^165^

#### 3.2.3 Other targeted biologics

B cells play an important part in the initiation and maintenance of RA inflammation through antigen presentation, secretion of proinflammatory cytokines, production of RFs, and T cell costimulation. Rituximab is a human‐mouse chimeric monoclonal antibody targeting the extracellular domain of CD20, which can initiate complement‐mediated lysis of B cells. After recognition by cytotoxic T cells through their Fc regions, it can induce antibody‐dependent cytotoxicity and promote B cell apoptosis, affecting the B cell response to antigens or other stimuli.^166^ Rituximab is mainly used to treat seropositive RA patients who are refractory to TNF‐α inhibitors, especially those with vasculitis and cryoglobulinemia.^167^ However, the safety of repeated rituximab treatment is still questioned, as some patients may be susceptible to serious infections and fatal progressive multifocal leukoencephalopathy.^147^ Clinical trials for two other CD20 monoclonal antibodies, ocrelizumab and ofatumumab, in the treatment of RA have been terminated due to adverse events.^168^

CD4^+^ T cells are key driving factors in synovial inflammation during RA. Activated T cells can express CTLA‐4, which interferes with the interaction between B7 and CD28, thereby reverting T cells to a resting state. Abatacept is a fusion protein of the extracellular domain of human CTLA‐4 and the Fc region of human IgG1, blocking T cell activation and exerting strong anti‐inflammatory effects.^169^, ^170^ Abatacept has better efficacy in patients with severe immunological abnormalities accompanied by autoantibodies and contributes to the improvement of RA‐associated interstitial pneumonia. The incidence of severe infections is significantly lower with abatacept compared to other biologics. Other T cell‐targeted drugs such as ALX‐0061, clazakizumab, and olokizumab are still under investigation.^171^

### 3.3 Small molecule DMARDs

Reversible protein phosphorylation by kinases and phosphatases is a fundamental mechanism of signal transduction. Kinases and phosphatases link the membrane events of ligand–receptor binding to calcium regulation, cytoskeleton rearrangement, gene transcription, and lymphocyte activation.

#### 3.3.1 JAK inhibitors

The important role of the JAK–STAT signaling pathway in inflammatory responses has been detailed previously. Tofacitinib, a pan‐JAK inhibitor, has been approved for the treatment of active RA when MTX is ineffective. Multiple clinical trials have demonstrated that tofacitinib provides sustained and significant improvement in symptoms and signs of RA, with ACR20 response rates of 50−60%, comparable to adalimumab. Moreover, the average change in mTSS was significantly better than MTX, indicating its effect on slowing down radiographic progression.^172^ However, the risks associated with herpes zoster, tuberculosis, and thromboembolism in RA patients with tofacitinib deserve attention.^173^ The selective JAK1/2 inhibitor baricitinib has also been approved for the treatment of RA. Other JAK inhibitors under development include selective JAK1 inhibitor GLPG0634 and ABT‐494, as well as selective JAK1/3 inhibitors VX‐509 and ASP015K.^174^

#### 3.3.2 Phosphodiesterase‐4 inhibitors

Phosphodiesterase‐4 (PDE4) is an enzyme responsible for degradation of intracellular cyclic adenosine monophosphate (cAMP). PDE4 inhibitors can significantly increase the level of cAMP, one of the most important second messenger in cellular signal transduction during autoimmunity and inflammation. cAMP can not only promote the upregulation of anti‐inflammatory factors such as IL‐10 through the activation of cAMP‐dependent protein kinase A, but also inhibit NF‐κB‐dependent TNF‐α secretion. In addition, PDE4 is also involved in the processes of adhesion molecule expression, chemotaxis, and degranulation in neutrophils.^175^ PDE4 inhibitors have been approved and used in psoriasis and psoriatic arthritis. Several compounds of PDE4 inhibitors have already been tested in clinical trials for RA. Although apremilast failed, the phase II clinical trial results of GRC4039 are still promising. Furthermore, Ibudilast has been confirmed to be able to reduce inflammatory mediators and inhibit disease progression in preclinical studies, making it a good candidate for RA clinical trials.^176^

## 4 EMERGING THERAPIES

Although a great progress has been made in the treatment of RA, no regimen could actually cure the disease, and almost all patients need lifelong therapy. Therefore, many emerging therapies are under exploration in preclinical and clinical studies.

### 4.1 IL‐23/Th17 pathway

Given the pathogenic role of IL‐17 in inflammation and tissue injury, targeting IL‐17 or its upstream IL‐23 in the treatment of autoimmune diseases has been explored long time ago. Currently, it has been confirmed that targeting the IL‐23/Th17 pathway has good efficacy in psoriatic arthritis, ankylosing spondylitis, and Crohn's disease. In a phase II clinical studies of secukinumab for RA, ACR20 reached 46%. Another IL‐17A inhibitor ixekizumab could rapidly improve clinical parameters in about one week. Though mechanistically rational, the effectiveness of IL‐17 inhibitor is overall moderate compared to TNF‐α inhibitors. In addition, the clinical trial results of AMG827, ABT‐122, and SCH‐900117 are also worthy of expectation.^177^

### 4.2 Sphingosine‐1‐phosphate receptor modulators

Sphingosine‐1‐phosphate receptor (S1P) is a kind of bioactive lipid, mainly derived from red blood cells and endothelial cells. S1P acts on S1P receptors (S1PR) for the regulation of cell growth, differentiation, and migration.^178^ Targeting the S1P/S1PR signaling axis reduces the trafficking of autoreactive lymphocytes and the Th17/Treg ratio, thereby controlling autoimmunity and inflammatory responses.^179^ Fingolimod, a S1PR1 inhibitor, has been approved for the treatment of multiple sclerosis. In a study of adjuvant‐induced arthritis, the S1PR1‐specific antagonist NIBR‐0213 can effectively prevent arthritis but may cause diffuse alveolar hemorrhage and pulmonary interstitial fibrosis.^180^ Another S1P/S1PR signaling axis modulator, LX2932, also does not reach the clinical endpoint of ACR20 response.^181^

### 4.3 Other promising targets

Immunotherapy targeting the programmed cell death protein 1 (PD‐1) pathway has proved to be effective against various cancers. However, inflammatory arthritis, a common immune‐related adverse events has arisen attentions. Considering its pivotal role in suppressing T cell activation, peresolimab, a humanized IgG1 monoclonal antibody designed to stimulate PD‐1, was demonstrated to be effective for RA in a phase 2a clinical trial.^182^ Many agents aiming at different cytokines and chemokines have been developed for RA treatment. For example, tadekinig alfa targeted for IL‐18,^183^ MOR103 targeted for GM‐CSF,^184^ and dekavil (an agonist of IL‐10)^185^ are all under clinical trials. Also, a clinical trial of E6011 (an anti‐CX3CL1 mAb) showed a promising role in active RA patients.^186^

An overwhelming success of JAK inhibitors has stimulated the development of medications with various kinases as targets. Bruton's tyrosine kinase (BTK) inhibitors have been approved for certain hematologic malignancies and are potential therapeutic agents for treating RA. Clinical trials for BTK inhibitor CC‐292 are ongoing.^187^ IL‐1 receptor‐associated kinases (IRAK‐4) mediates pathogen recognition and cytokine release such as IL‐1, IL‐6, and TNF. Furthermore, the activity of IRAK‐4 kinase regulates Th17 differentiation in RA. PF‐06650833 targeted for IRAK‐4 is also quite promising in future RA treatment.^188^

With the clarification of epigenetic mechanisms in RA, key enzymes that regulate important biological processes such as DNA methylation, RNA m^6^A, and histone modification might become potential therapeutic targets for RA. The functions of azacitidine targeted for DNMT,^189^ anacardic acid and MB‐3 targeted for HAT,^96^, ^190^ GSK‐J4 targeted for KDM,^191^ MS‐275 targeted for HDAC,^192^ I‐BET151 targeted for BRD4^193^ have been verified in animal studies.

Due to the abnormal metabolism in FLS and T cells of RA patients, many researchers have moved their attentions to metabolic medications. For example, metformin can selectivity inhibit mitochondrial respiratory chain complex I and decrease NADPH oxidase activity, thus leading to a remarkable decrease in ROS production. Recently, a study presented its potential impact in the treatment of SLE according to its metabolic properties and the inhibition of NETosis.^194^ Therefore, a phase 2 clinical trial for MTX/metformin versus MTX alone on the decrease of RA activity is recruiting. More details could be found in Table 3.

**TABLE 3:** Medications approved, on trial or preclinical in RA.

Drug	Type	Targets	Comments	References
**Approved**				
Methotrexate	csDMARD	AICAR and adenosine	First‐line “anchor” drug Slow acting, and toxic to many organs	^155^
Leflunomide	csDMARD	DHODH	Most commonly used substitutes for MTX Slow acting, and toxic to organs	^156^
Sulfasalazine	csDMARD	Multiple ways	Usually a part of combination therapy	^157^
Hydroxychloroquine	csDMARD	Multiple ways	Usually a part of combination therapy Slow acting, and toxic to eyes	^158^
TNF inhibitors	bDMARD	TNF‐α	Used as early as possible in patients with poor prognostic factors More infection with tuberculosis	^159^
IL‐6R inhibitors	bDMARD	IL‐6R	Especially suitable for refractory RA	^163^
Anti‐CD20 monoclonal antibody	bDMARD	B cell	Especially suitable for RA patients with vasculitis and cryoglobulinemia More infection with viruses	^167^
CTLA‐4 analogue	bDMARD	T cell	Especially suitable for RA patients with immunodeficiency and interstitial pneumonia	^170^
JAK inhibitors	tsDMARD	JAK	As effective as bDMARDs More infection with herpes zoster	^172^
**On trial**				
GRC4039	tsDMARD	PDE4	Approved in psoriasis and psoriatic arthritis, but moderate effective in RA	^176^
Secukinumab and ixekizumab	bDMARD	IL‐17	Approved in psoriasis and psoriatic arthritis, but moderate effective in RA	^177^
NIBR‐0213 and LX 2932	tsDMARD	S1PR	Reducing trafficking of autoreactive lymphocytes, but may cause severe pulmonary adverse events	^180^, ^181^
Peresolimab	bDMARD	PD‐1	Proved to be effective in RA with respect to American College of Rheumatology (ACR) 20, but not ACR50 and ACR70	^182^
Tadekinig alfa	bDMARD	IL‐18	Proved to be effective in adult‐onset still's disease	^183^
MOR103	bDMARD	GM‐CSF	Well tolerated with preliminary evidence of efficacy in a phase2 trial	^184^
Dekavil	bDMARD	IL‐10	Good response in a Ib trial	^185^
E6011	bDMARD	CX3CL1	Well tolerated with response rates of ACR20 between 40 and 70%	^186^
CC‐292	tsDMARD	BTK	Proved to be effective in chronic lymphocytic leukemia	^187^
PF‐06650833	tsDMARD	IRAK‐4	Proved to be effective in systemic lupus erythematous	^188^
Metformin	Metabolic drug	Glucose metabolism	Already determined effective in systemic lupus erythematous	^193^
**Preclinical**				
Azacitidine	Epigenetic regulator	DNMT	Proved to be effective in animal models	^189^
Anacardic acid and MB‐3	Epigenetic regulator	HAT	Proved to be effective in animal models	^96^, ^190^
GSK‐J4	Epigenetic regulator	HMT	Proved to be effective in animal models	^191^
MS‐275	Epigenetic regulator	HDAC	Proved to be effective in animal models	^192^
I‐BET151	Epigenetic regulator	BET	Proved to be effective in animal models	^193^
ERG240	Metabolic regulator	BCAT1	Proved to be effective in animal models	^150^

## 5 CONCLUSION AND PROSPECTIVE

The past decades have witnessed the rapid development of RA pathogenesis and treatment, but some important issues remain unresolved. First, curing the disease depends on the recognition of etiology, which is largely unclear yet. With more studies focused on the pathogenesis of RA, especially studies on epigenetics and immunometabolism, an interference during preclinical stage might offer an opportunity to stop immune responses and disease onset. Another challenge is a lack of the rationale of personalized and precision medicine.^195^ Although there has been much progress in the development of therapeutic medications, current treatment still follows a trial‐and‐error strategy because we are unable to predict the most effective drug for individual patients. Previous studies have shown that high C‐reactive protein and low body mass index are associated with a better response of TNF‐α inhibitors, while smoking has the opposite effect.^196^, ^197^, ^198^ Muskardin et al.^199^ found that a ratio of IFN‐β to IFN‐α higher than 1.3 in serum could predict no response to TNF‐α inhibitors. According to synovial biopsy, Dennis et al.^200^ demonstrated that synovitis in RA could be divided into four subgroups, lymphoid, myeloid, low inflammatory and fibroid, among which, a myeloid gene expression pattern in synovial tissue associated with good response to TNF‐α inhibitor therapy. Anyway, precision medicine is still an emerging area in RA. As for anti‐TNF‐α inadequate responder, for patients with low or absent B cell lineage expression signature in synovial tissue, tocilizumab is more effective than rituximab, revealed by R4RA trial. Also, this study demonstrated that RNA sequencing‐based stratification of RA synovial tissue showed stronger associations with clinical responses compared with histopathological classification.^201^, ^202^ Therefore, more genetic–pathological–clinical studies are needed to fuel continued progress toward precision medicine in this field.

Furthermore, new strategy is also under requirement in terms of regenerative medicine and tissue engineering. More than 10 clinical trials based on MSCs therapy for RA have been completed, using autologous or allogeneic MSCs from different sources.^203^ However, there is only low‐level evidence that MSCs are symptom‐relieving, but no data supporting a decrease of ACR20 or a repair of cartilage in RA. Recently, the inspiring success of chimeric antigen receptor T‐cell (CAR‐T) therapy in SLE has been reported.^204^ Considering the good response to rituximab in refractory RA, CAR‐T therapy depleting CD19^+^ B cells are very likely to bring us into another era of RA treatment. And more customized therapy with other pathogenetic targets of CAR‐T will be developed and tested in the future. For example, in a proof‐of‐concept study, Zhang et al.^205^ developed an engineered T cells with antifluorescein isothiocyanate (FITC) CAR to eliminate autoreactive B cell subsets recognizing citrullinated peptide epitopes in RA, which is quite promising.

To conclude, RA is a complex clinical entity with pathogenesis involving abnormal immune response, inflammatory pathways, genetics and epigenetics, and immunometabolism regulation. As discussed in this review, our insights into the pathogenesis of RA have reached a new altitude. Whether these immunologic, epigenetic, and metabolic targets could be transformed into predictive factors or clinical interventions need more innovative study designs. Numerous therapeutic options make RA, a highly disabling disease, become controllable, especially biologic DMARDs and JAK inhibitors. Although inflammation extinguished and tissue damage decelerated, drug‐free remission is still far from reach in RA. With deeper mechanism uncovered and stronger targets clarified, an attempt to cure or at least prevention for RA would be achieved in the near future.

## AUTHOR CONTRIBUTIONS

Xingguang Liu, Ying Gao, and Yunkai Zhang conceptualized this review. Ying Gao and Yunkai Zhang drafted the manuscript. Xingguang Liu, Ying Gao, and Yunkai Zhang revised the manuscript. All authors approved the final manuscript.

## CONFLICT OF INTEREST STATEMENT

The authors declare no conflict of interests.

## ETHICS STATEMENT

Not applicable.

## Data Availability

Not applicable.
